# Half versus full vacuum suction drainage after modified radical mastectomy for breast cancer- a prospective randomized clinical trial[ISRCTN24484328]

**DOI:** 10.1186/1471-2407-5-11

**Published:** 2005-01-27

**Authors:** Chintamani, Vinay Singhal, JP Singh, Anju Bansal, Sunita Saxena

**Affiliations:** 1 Department of Surgery, Vardhman Mahavir Medical College Safdarjang Hospital New Delhi – India; 2 Vardhman Mahavir Medical College Safdarjang Hospital New Delhi-110023 – India; 3 Tumor Biology Lab Indian Council Of Medical Research (ICMR) New Delhi – India

## Abstract

**Background:**

Suction drains are routinely used after modified radical mastectomy and are an important factor contributing to increased hospital stay as the patients are often discharged only after their removal. Amongst various factors that influence the amount of postoperative drainage, the negative suction pressure applied to the drain has been reported to be of great significance. While a high negative suction pressure is expected to drain the collection and reduce the dead space promptly, it may also prevent the leaking lymphatics from closing and lead to increased drainage from the wound. Against this background a prospective randomized clinical study was conducted to compare the amount and duration of drainage between a half negative suction and full vacuum suction drainage in patients following modified radical mastectomy. The associated postoperative morbidity was also compared between the two groups.

**Methods:**

85 FNAC (fine needle aspiration cytology) proven cases of locally advanced breast cancer were randomized. (Using randomly ordered sealed envelops, which were opened immediately before the closure of the wound) in to 50 patients with full vacuum suction (pressure = 700 g/m2) and 35 cases in to half vacuum suction drainage (pressure = 350 g/m2) groups. The two groups were comparable in respect of age, weight, and technique of operation and extent of axillary dissection. Surgery was performed by the same surgical team comprising of five surgeons (two senior and three resident surgeons) using a standardized technique with electrocautery. External compression dressing was provided over the axilla for first 48 hrs and following that patients were encouraged to do active and passive shoulder exercises. The outcomes measured were postoperative morbidity and the length of hospital stay.

Statistical methods used: Descriptive studies were performed with SPSS version 10 and group characteristics were compared using student t-test.

**Results:**

Half vacuum suction drains were removed earlier than the full suction vacuum suction drains. There was no significant difference in the incidence of seroma formation in the two groups and there was a significant reduction in the total hospital stay in patients with half vacuum suction drainage systems as compared to the full suction drainage group (p < 0.001) without any added morbidity.

**Conclusions:**

Half negative suction drains provide an effective compromise between no suction and full or high suction drainage after modified radical mastectomy by reducing the hospital stay and the post operative morbidity including post operative seromas.

## Background

Suction drainage in the management of mastectomy patients was used for the first time in 1947 [1] and has been found in various studies superior to other methods of fluid evacuation to minimize the dead space. The mechanism proposed is that the suction helps skin flaps to adhere to the chest wall and axilla sealing off all the leaking lymphatics[2,3]. This reduces the incidence of post-operative seromas, hematoma formation and flap necrosis, which are, recognized complications of modified radical mastectomy [2,3]. When no postoperative suction drains were used the incidence of seromas was found to be unacceptably high in various studies [4]. Prolonged drainage on the other hand, may increase the hospital stay and increase the risk of infection by allowing retrograde migration of bacteria [4]. Indiscriminate or premature withdrawl of postoperative drains irrespective of the amount of fluid drained may be accompanied by an increase in the incidence of axillary seromas [4-6]. If kept for longer periods it has been observed that drain itself might contribute to increased drainage and the risk of infection in addition to the increased hospital stay resulting in to wasteful utilization of the hospital resources. The amount of postoperative drainage is influenced by various factors like the clinical profile of the patient including the body mass index, extent of axillary lymph node dissection, number of lymph nodes dissected, use of elctrocautery, co morbid conditions and also the *negative pressure *on the suction drain [4-10]. The amount of postoperative fluid drained has been found to be significantly influenced by the negative pressure on the suction drainage. While the negative suction drain is logically expected to drain the fluid, a high negative suction drain may prevent the leaking lymphatics from sealing off thus leading to prolonged drainage leading to increased hospital stay [4]. The present prospective randomized clinical trial compared the postoperative wound drainage in patients with full suction drain (high) and those with half vacuum drainage system (low).

The study also compared the drain volume, average hospital stay and postoperative morbidity between full vacuum and half vacuum suction groups.

## Methods

The study was conducted in one surgical unit of a tertiary care center over a period of two years.85, FNAC (fine needle aspiration cytology) proven cases of locally advanced breast cancer were randomized (using randomly ordered sealed envelops, which were opened immediately before the closure of the wound) into full vacuum suction (pressure = 700 g/m2) group – (A) and 35 cases into half vacuum suction (pressure = 350 g/m2) group – (B). The two groups were comparable in respect of age, weight and type of operation i.e. modified radical mastectomy (MRM). Following complete routine and metastatic work up, all patients received three cycles of Neoadjuvant chemotherapy (NACT) using CAF regime (Cyclophosphamide, Adriamycin, 5-Fluorouracil) and underwent Patey's modified radical mastectomy after three weeks of the last cycle. Surgery was performed by the same surgical team comprising of five surgeons (two senior and three resident surgeons) using a standardized technique with electro cautery. Axillary dissection was done up to level- III in all the cases. The boundaries of axillary dissection were defined by superior limit as the posterolateral border of the Pectoralis major muscle and axillary vein, medial limit being clavipectoral fascia or Hallstead's ligament, lateral limit as the anterior border of lattismus dorsi and the inferior limit being the angular vein joining the thoracodorsal vein. The long thoracic and thoracodorsal nerves were identified, dissected and preserved. Two silicone tube drains (12Fr) (one axillary and pectoral) were inserted in all the patients. All resected specimens were examined and the lymph nodes dissected, counted and assessed histo-pathologically for metastases. No patient received intra-operative blood transfusion. Both the drains were connected to a single 600 ml suction bottle (Romovac -Romson). In-group A (n = 50), drainage was performed using complete vacuum negative suction (700 g/m2) and in-group B (n = 35) with half vacuum suction drainage (350 g/m2). The pressure was also measured by attaching a manometer to the exit opening of the drainage bottle. The two groups were comparable with respect to age, weight (body mass index), type of operation indicating the success of randomization (Table. 1). The drain was emptied every 24 hours to reset suction at the respective pressures and to measure the daily drain out put. External compression dressing was provided over the axilla for first 48 hrs and following that the patients were encouraged to do active and passive shoulder exercises. The outcomes measured were morbidity and the length of hospital stay. The total drain output was measured and recorded daily in both the groups, the drains were removed once the output was less than 30 ml in 24 hrs and the patients were discharged on the same day. The mean total drain output was measured in each group and compared. The mean hospital stay in both the groups was calculated and compared. The associated morbidity in the form of seroma formation, flap necrosis and wound infection during the postoperative period was recorded and compared in both the groups

### Statistical methods used

Descriptive studies were performed with SPSS version 10 and group characteristics were compared using student t-test (Tables. 2&3).

## Results

1. Half vaccum suction drains were removed earlier than full vacuum suction drains without any significant addition to the postoperative morbidity.

2. The use of half vacuum drains after modified radical mastectomy reduced the hospital stay significantly without any increase in the postoperative morbidity.

3. The high negative suction is an important contributory factor to the amount of drainage following breast surgery along with axillary dissection. While negative suction helps in prevention of postoperative seroma formation, a high negative suction may affect adversely by increasing the amount and duration of drainage. This probably is on account of persistent drainage from the lymphatics, which do not close due to high negative suction.

## Discussion

Seroma formation is the most frequently observed early complication after breast and axillary surgery. The use of closed suction drainage is a common practice that has been shown to reduce the incidence of seroma formation [1-6]. These drains are generally removed once the lymph production falls to less than 35–50 ml/24 hours, a level generally reached between 3–17 days after surgery [1]. The length of postoperative axillary drainage is a major cause of morbidity after axillary dissection as the patients are usually discharged once the drains are removed. The patients with suction drains in situ are normally managed in the hospital (although some authors advocate discharge with the drains in situ)[13]. Migration of bacteria along these drains has also observed to increase the risk of infection if the drains stay in situ for a long time [7]. Early or premature removal however has been found to be associated with an unacceptably high incidence of seroma formation and its continuation until fluid discharge is acceptably low leads to a prolonged stay in the hospital, which has a bearing on the cost of surgical management of breast cancer [1,11-13]. Shortening the hospital stay has been shown to be an effective way of reducing the costs in the case of surgery for breast cancer and axillary drains are the main obstacles in achieving it [1,8-10]. To reduce the hospital stay after MRM, early discharge with the drains in situ has been reported but discharging patients with drains in situ has an inherent difficulty faced by the patients in management of drains besides higher incidence of wound infection [13,14]. The other disadvantages are discomfort for the patients, with difficulties undressing or using the toilet. It may be feasible with patients of higher cultural and social standing, but not all the patients have the required background. In a third world country where the patients are poor, uneducated coming from far and remote areas with limited medical facilities, there is an added difficulty in management of the drains away from the hospital. As most of our patients come from far flung rural areas with limited education, poor medical and communication facilities they were managed indoors until the drains were removed.

There are other solutions proposed for prevention or reduction of fluid accumulation and early discharge after axillary dissection e.g by Patrek et al [15,16] where several parallel drains were used. Suture obliteration of axillary space under skin flaps with sutures to the chest wall, approximation of the pectoralis major and the latissimus dorsi muscle in the form of axillary padding has been suggested by some authors [9,17]. The incidence of seroma formation had reduced but the length of drainage was not specified in these studies. Further more suture approximation of the muscles may limit movement of the arm leading to shoulder dysfunction. Harada et al [14] used fibrin glue in rats to occlude transected lymph channels and obliterate the subcutaneous cavity.

The association of seroma formation with large amounts of drainage before removal of the drain has already been established [18-20]. In one study it was observed that when the amount of fluid drained before removal of the catheter was less than 250 ml in three days no seromas developed and they concluded that it is safe to remove drains if the total amount of fluid drained during the first postoperative days is low. Yii et al [20] reported that removal of drains after 48 hours did not result result in seroma formation if the total amount of fluid drained before removal was less than 150 ml

### Proposed factors contributing to the increased drainage and seroma formation [2,3,8-15]

Patrek et al [15] examined 13 factors influencing fluid drainage. Only two (a large number of positive lymph nodes and previous biopsy) predicted greater drainage.

1.**Body mass index**. A significant linear relation exists between BMI and increased seroma formation was reported by (Boonman et al)

2. **Technique**: Use of elctrocautery has been reported to be associated with increased incidence of seroma formation as compared to cold knife. It has also been reported that tissue ligation around the axillary vein rather than mere transection with knife or diathermy may reduce the amount of postoperative discharge, the technique was followed in the presented study [8].

3 **Drains themselves **encourage drainage by stimulating tissue reactions or by suction [20]

4.**Early shoulder exercises **have been implicated but were not observed to be a factor in the present study [10,11] Although early mobilization of the shoulder did not increase fluid discharge in various studies but it was reported to be an additional factor leading to increased drainage after axillary dissection [10,11].

5 **The negative suction **applied may prevent the lymphatics from closing leading to continuous leakage and discharge [18].

6.**Extent of axillary dissection**. More seromas were seen when more lymph nodes were dissected from the axilla. The higher lymph node yield may well be an indirect measure of more extensive dissection performed. [3]. The drainage may also reflect the damage to the lymph vessels and therefore the number of lymph nodes dissected may have a bearing on the amount of drainage. In our study also, it was observed that patients with higher lymph node yield had a higher volume and duration of drainage although it was not found to be significantly different in both the groups because they were matched in all respects except the negative suction pressure of the drainage.

### Negative suction and the drainage

It is an accepted fact that negative suction prevents seroma collection and helps in the adherence of the walls of the axilla thus reducing the dead space and allowing the lymphatics to close. High negative suction pressure generated by the drain can maintain lymph drainage by a negative pressure gradient [18]. It is also reported that the high negative suction pressure does not allow the lymphatic channels to close leading to continuous drainage and a higher incidence of seroma formation [18-20]. There are studies to suggest that high negative suction may be beneficial in the sense that the amount of drainage would be more thus allowing an early adherence of walls of the axilla to the chest wall and reduction in the seroma formation [18]. However in the present study it was observed that high suction caused prolonged drainage, which can possibly be explained by the hypothesis that high negative suction may not allow, leaking lymphatics to close. Therefore no suction or high suction drainage both may contribute to the same result that is higher incidence of seroma formation and longer hospital stay. To strike a balance between not having suction at all and having a very high or full negative suction, half negative suction drainage was used in the present study to achieve a shorter hospital stay without any increase in the rate of post operative seroma formation. The external compression dressings in the first forty-eight hours perhaps helped in the adherence of the flaps and reduction of dead space without compromising on the shoulder mobility. This was found to effectively reduce the Hospital stay and also did not increase the postoperative morbidity as compared to high (full) negative suction group.

## Conclusions

Reducing the negative suction pressure applied to the drain (making it half suction) along with external compression dressings applied for first 48 hours can significantly reduce drainage from the axilla following modified radical mastectomy without increasing the incidence of seroma formation as was observed in this randomized prospective clinical study. The hospital stay was reduced considerably compared to a matched group with full suction drain (p < 0.001). Half suction drain following axillary dissection in patients with carcinoma breast may thus be recommended as an effective approach to reducing the hospital stay and the cost of treatment without adding to the morbidity.

## Competing interests

The author(s) declare that they have no competing interests.

## Authors' contributions

CM was the surgeon in charge of the project and prepared the study design. VS was the first surgical assistant and did the data processing and statistical analysis. JP, the second surgical assistant assisted in the preparation of the manuscript. SS the Director of ICMR and AB the research officer, Tumor Biology Lab ICMR were responsible for the histopathological analysis and contributed to the preparation of the manuscript.

## Pre-publication history

The pre-publication history for this paper can be accessed here:



## Figures and Tables

**Table 1:** 

	Full vaccum group n = 50	Half vaccum group n = 35	p- value
Mean Age (years)	49.54 (S.D = 9.821)	46 (S.D = 7.580)	-
Mean Weight (kg)	54 (S.D = 6.543)	55 (S.D = 5.285)	-
No. Of lymph nodes resected (mean)	12.5 (S.D = 1.619)	12 (S.D = 1.328)	-
% With positive lymph nodes	28%	30%	-
NACT regime	CAF	CAF	-
Surgery	MRM	MRM	-
Mean volume drained (ml)	525 (S.D = 66.282)	325 (S.D = 39.612)	<0.001
Mean hospital stay (days)	10.8 (S.D = 1.603)	6 (S.D = 1.414)	<0.001
BMI	21.6 (S.D = 2.347)	21.7 (S.D = 1.800)	-
Morbidity			
Flap necrosis	1	1	NS
Wound infection	4	3	NS
Seroma formation	2	1	NS
Seroma aspirations required	None	None	-

**Table 2:** Non parametric test

	Group	n	Mean Rank
Drain vol	1.00	50	60.49
	2.00	35	18.01
	Total	85	

**Table 3:** Mann Whitney U test

	Drain vol
Chi square	61.044
df	1
Asymp. Sig.	<0.05
